# The real identity of *Leptodira nycthemera* Werner, 1901 from Ecuador: a junior synonym of *Oxyrhopus petolarius* (Linnaeus, 1758) (Serpentes, Dipsadidae)

**DOI:** 10.3897/zookeys.506.9074

**Published:** 2015-06-01

**Authors:** João Carlos Lopes Costa, Christoph Kucharzewski, Ana Lúcia da Costa Prudente

**Affiliations:** 1 Museu Paraense Emílio Goeldi, Coordenação de Zoologia, Programa de Pós-Graduação em Zoologia, Av. Perimetral, 1901, Bairro Terra Firme, 66077-530, Belém, PA, Brazil; 2 Museum für Naturkunde - Leibniz-Institut für Evolutions- und Biodiversitätsforschung, Invalidenstraße 43, 10115 Berlin, Germany; 3 Museu Paraense Emílio Goeldi, Coordenação de Zoologia, Av. Perimetral, 1901, Bairro Terra Firme, 66077-530, Belém, PA, Brazil

**Keywords:** *Leptodeira annulata annulata*, new synonym, *Oxyrhopus petolarius*

## Abstract

*Leptodira nycthemera* Werner, 1901, was described from a specimen collected in Ecuador. No information on the holotype was published after its description. In the most recent review of *Leptodeira*, *Leptodira nycthemera* was considered to be a synonym of *Leptodeira annulata annulata*, although the author emphasized that the holotype was lost and did not include the pholidotic data from the original description in his account of *Leptodeira annulata annulata*. Since this review, a number of authors have accepted this synonymy. Recently, analyzing specimens of *Leptodeira* in the Museum für Naturkunde, Berlin, Germany, we discovered the holotype of *Leptodira nycthemera*. This holotype is re-described here, and its correct identity is determined. Based on the analysis of meristic characters and the color of the holotype, we recognize *Leptodira nycthemera* as a junior synonym of *Oxyrhopus petolarius*.

## Introduction

*Leptodira nycthemera* was described by Werner (1901), based on a specimen collected in Ecuador by the German entomologist Richard Haensch. However, this original description lacks sufficient information for the definition of the species. The exact type locality also remains unknown, given that Haensch and his companion Edmund Schmidt travelled through large parts of cis- and trans-Andean central Ecuador (Haensch 1903, Racheli and Racheli 2001, 2003).

Subsequently, Werner (1913) described *Leptodira dunckeri* without defining a precise locality, which might be Mexico or Venezuela, and presented a key to the species of the genus that included *Leptodira nycthemera*. Müller (1923) described *Leptodira weiseri* from Argentina, which differed from *Leptodira nycthemera* by having a subocular scale, reduced number of ventral and subcaudal scales, in addition to a very distinctive color pattern. *Leptodira weiseri* was subsequently considered to be a synonym of *Oxyrhopus rhombifer bachmanni* (Weyenberg, 1876) by Bailey (1970). Werner (1924) presented the second part of a survey of the snakes of the family Colubridae, including identification keys. As a valid species, *Leptodira nycthemera* is distinguished from all other Neotropical *Leptodira* taxa by the presence of an undivided anal scute.

Amaral (1930a) redefined *Leptodira dunckeri*, *Leptodira nycthemera*, and *Leptodira weiseri* as synonyms of *Leptodeira annulata*. However, while recognizing *Leptodira dunckeri* and *Leptodira weiseri* as junior synonyms of *Leptodeira annulata annulata* (Linnaeus, 1758), Amaral (1930b) did not refer specifically to *Leptodira nycthemera*.

In a review of the *Leptodeira* species of North America, Dunn (1936) presented a list of synonyms of *Leptodeira annulata annulata*, in which *Leptodira nycthemera* was not mentioned, referring only to *Leptodira dunckeri* as a junior synonym of *Leptodeira septentrionalis maculata* (Hallowell, 1861). Taylor (1938), revising *Leptodeira* from Mexico, did not mention *Leptodira nycthemera*, but recognized *Leptodira dunckeri* as a valid species.

The name *Leptodira nycthemera* reappeared in the most recent review of *Leptodeira*, presented by Duellman (1958). In this case, *Leptodira nycthemera* was considered to be a junior synonym of *Leptodeira annulata annulata*, although the author emphasized the fact that the holotype had been lost, and the pholidotic data from the original description were not included in the account of *Leptodeira annulata annulata*. Subsequently, a number of authors (Peters 1960, Peters and Orejas-Miranda 1970, Kornacker 1999, Wallach et al. 2014) accepted the synonymy of *Leptodira nycthemera*, even though no further information on the locality of the specimen or the morphological characteristics of the taxon have been provided, until now.

Recently, we discovered the *Leptodira nycthemera* holotype during the analysis of the *Leptodeira* specimens at the Museum für Naturkunde in Berlin, Germany. This specimen is redescribed here, and its taxonomic status is determined.

## Material and methods

Measurements of the specimens are presented in millimeters, and were taken with a digital caliper and flexible ruler. The measurements of the head and cephalic scales have a precision of 0.1 mm, and those of the SVL and tail, a precision of 1 mm. The head length was defined as the distance between the rostral and the angle of the jaws. Head width is the widest point of the head at the level of the temporal scales.

The cephalic scales were counted on both sides (right/left) of the head and body (Peters 1964). Scales were measured based on the largest dimensions of the visible portion. Ventral scales were counted according to Dowling (1951a) and the formula for the reduction of the dorsal scale row was based on Dowling (1951b). We determined the sex of the specimen by the presence or absence of a hemipenis, inspected visually through a ventral incision at the base of the tail.

## Results

The type specimen of *Leptodira nycthemera* Werner, 1901, is currently housed in the Museum für Naturkunde in Berlin, Germany, under catalog number ZMB 16596.

**Figure 1.**
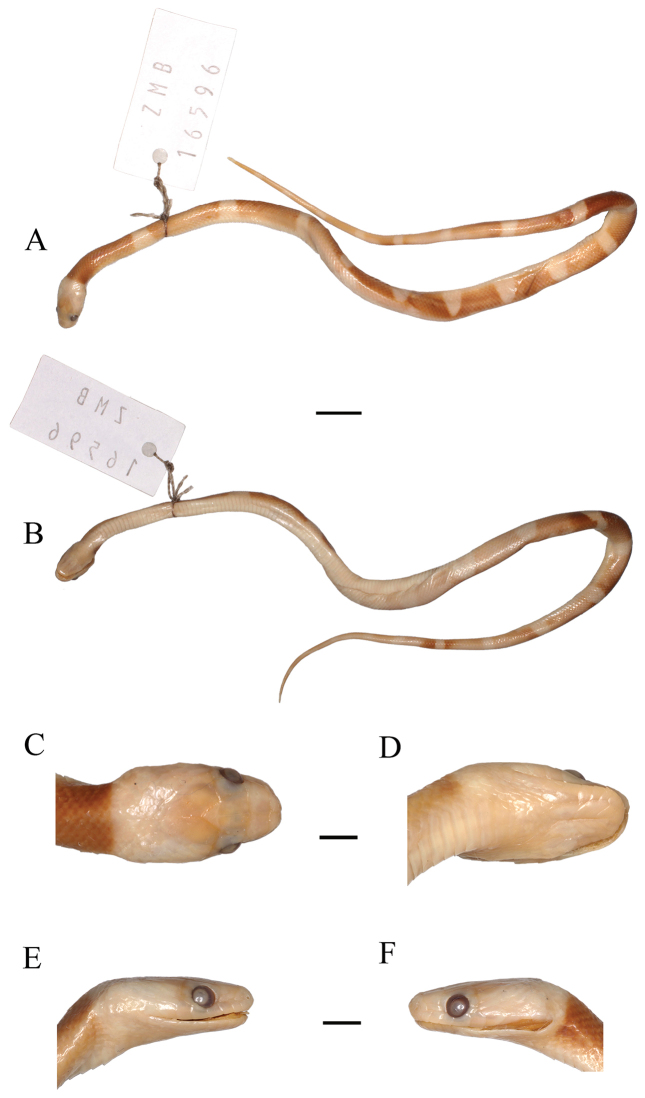
Holotype of *Leptodira nycthemera* Werner, 1901 (ZMB 16596). **A** dorsal view of the body **B** ventral view of the body **C** dorsal view of the head **D** ventral view of the head **E** right lateral view of the head, and **F** left lateral view of the head. Scale bar: 10 mm (**A, B**), 2.5 mm (**C–F**).

**Redescription**: The holotype is a juvenile female (Figure 1) as indicated by the presence of a distinctly recognizable umbilicar scar on ventral scales 174–175. The specimen is in a good state of preservation, with the following characters: **Folidosis**: loreal approximately twice as long as high; internasals approximately half the length of the prefrontals; frontal longer than wide; parietals longer than wide; preoculars 1 / 1, in contact with frontal; postoculars 2 / 2; supralabials 8 / 8, the fourth and fifth in contact with the orbit, sixth and seventh of approximately the same size; temporals 2 + 3 / 2 + 3; infralabials 10 / 10 [a lesion on left side of infralabial region did not affect the count of this character], first to fifth in contact with the anterior genials, fifth and sixth in contact with the posterior genials; two pairs of genials of nearly the same size; dorsal scales smooth, apical pits absent, 19 / 19 / 17 dorsal rows, reduction by fusion of dorsal scale rows 3 + 4, according to the formula:


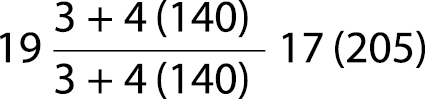
;

vertebral row not enlarged; 2 preventrals + 205 ventrals; anal scute undivided; 101 / 101 subcaudals + terminal spine. **Morphometric data**: Snout-vent length (SVL) = 200 mm; tail length = 64 mm; head length = 10.7 mm; head width = 6.6 mm; head height = 3.7 mm; horizontal eye diameter = 1.75 mm; distance from anterior margin of eye to nostril = 2.5 mm; frontal length = 3.4 mm; frontal width = 3.1 mm; parietal length = 4.2 mm; parietal width = 3.2 mm; anterior genials= 2.6 mm; posterior genials= 2.3 mm. **Proportions**: Ratio of tail length to total length = 32.5 %; ratio of head length to SVL= 5.35 %. **Color pattern in preservative**: The head is somewhat discolored but it is still possible to observe a dark area, as mentioned in the original description, which forms a hood covering the rostral, internasal, pre-frontal, frontal, and parietal scales; symphyseal and infralabials gray; occipital area white, starting at the supralabials, through the margins of the posterior parietal, and all occipitals; well preserved coloration of the body and tail, with clearly visible pigmentation, body with dark (black) bands, which extend to the edge of the ventral scales and are separated by light (white) bands; a pattern comprised of 12 + ½ black bands on the body and 6 + ½ on the tail, the bands merge starting at the fourth dorsal blotch forming a zigzag pattern, the dark bands are longer on the anterior portion of the body, and the three first are 19, 17, and 16 scales long on the vertebral line, respectively; ventrals and subcaudals scales are lightly colored (cream in preservative).

## Discussion

The holotype presents the typical characters of species of the genus *Oxyrhopus* (Bailey 1970, Duellman 1958, 1978, Peters 1960, Savage 2002, Lynch 2009), including: (i) the absence of apical pits (*vs.* two in *Leptodeira*), (ii) 2 + 3 temporal scales (*vs.* 1 + 2 in *Leptodeira*), (iii) undivided anal scute (*vs.* divided in *Leptodeira*), (iv) lateral reduction of the dorsal scale rows (*vs.* vertebral or paravertebral reduction in *Leptodeira*), (v) black banding pattern that reaches the ventral scales (*vs* small saddle-shaped or ovoid blotches, reaching only the sixth or seventh dorsal row in the *Leptodeira* specimens from Ecuador). This diagnosis allowed us to exclude *Leptodira nycthemera* from the genus *Leptodeira*.

The only species of *Oxyrhopus* from Ecuador that has the same color pattern and pholidosis as *Leptodira nycthemera* is *Oxyrhopus petolarius* (Linnaeus, 1758). A number of characters of the holotype are consistent with or within the range of the data presented for female *Oxyrhopus petolarius* by Duellman (1978) and Lynch (2009). These are (i) the high number of ventrals (191–225) and subcaudals (77–112), (ii) the number of bands on the body (11 ½–13 ½), (iii) preocular contacting the frontal, and (iv) juvenile individuals of *Oxyrhopus petolarius* (< 300 mm body length) have a black head, white occipital region, black bands on the body wider than the light ones, and dislocated black bands in the dorsal midline, adjacent to the middle of the body, forming a zigzag pattern.

Based on the analysis of meristic characters and the color pattern of the redescribed holotype, we recognize *Leptodira nycthemera* as a junior synonym of *Oxyrhopus petolarius*. *Oxyrhopus petolarius* has the most ample geographic distribution of the species of the genus, occurring from Veracruz, on the Atlantic slope of Mexico, and the Pacific slope of Costa Rica, through Central America, to western Equator, and throughout northern South America, including Bolivia, Brazil, Ecuador, and Peru (Savage 2002). Due to this wide distribution and the morphological variation found in *Oxyrhopus petolarius*, three subspecies are recognized – *Oxyrhopus petolarius digitalis*, *Oxyrhopus petolarius petolarius*, and *Oxyrhopus petolarius sebae*. These forms are differentiated by the number of dorsal blotches on the body, length of light bands on the posterior region of the body, and by the contact between postocular and frontal scales (Bailey 1970). However, Lynch (2009) identified inconsistencies in these characters, recognizing the need for more systematic studies of this geographical variation in order to elucidate the status of these taxa. For this reason, we have chosen to allocate *Leptodira nycthemera* only to the species level.

The taxonomy of *Oxyrhopus petolarius* is also subject to some controversy (see Savage 2011). Because of this, we have opted to follow Savage (2011) in using *Oxyrhopus petolarius* as the valid species name, rather than *Oxyrhopus petola*.

## Acknowledgments

We thank D. T. Feitosa and M. G. Pires, for providing information on the holotypes, J. F. Sarmento for helping with the herpetology collection at the Museu Emilio Goeldi, and F. Tillack, curator of the Herpetology Collection at the Museum of Natural History Leibniz, Institute for Research on Evolution and Biodiversity at the Humboldt University of Berlin, for the photographs. We are very grateful for the careful reviews of two anonymous referees. We are also grateful to Isabela Brcko for helpful suggestions on this manuscript. We thank S. Ferrari for improving the English writing. J. C. L. C. is supported by stipends from Conselho Nacional de Desenvolvimento Científico e Tecnológico (CNPq, process number PROTAX 140142/2011-8), and his research and that of A. Prudente is supported by CNPq (process number PROTAX 562171/2010–0; Pq 308950/2011-9; 305475/2014-2).
